# Development and validation of a combined glycolysis and immune prognostic signature for lung squamous cell carcinoma

**DOI:** 10.3389/fgene.2022.907058

**Published:** 2022-09-30

**Authors:** Qiang Huang, Shan Yang, Hao Yan, Hong Chen, Yuzhu Wang, Yang Wang

**Affiliations:** ^1^ Department of Respiratory and Critical Care Medicine, Chengdu Second People’s Hospital, Chengdu, China; ^2^ Department of Rehabilitation Medicine, Chengdu Sixth People’s Hospital, Chengdu, China; ^3^ National Cancer Center, Cancer Hospital, Chinese Academy of Medical Sciences and Peking Union Medical College, Beijing, China

**Keywords:** lung squamous cell carcinoma, glycolysis, immune, gene signature, prognosis

## Abstract

**Background:** The involvement of glycolysis in the regulation of the tumor immune microenvironment has become a novel research field. In this study, the specific functions and clinical significance of glycolysis-related genes (GRGs) and immune-related genes (IRGs) were systematically characterized in lung squamous cell carcinoma (LUSC).

**Methods:** We evaluated the prognostic value, interactions, somatic mutations, and copy-number variations of GRGs and IRGs in LUSC from a dataset of The Cancer Genome Atlas (TCGA). An integrated glycolysis–immune score (GIS) model was generated by random forest algorithm and stepwise Cox regression analysis. The predictive power of the GIS was examined by survival analysis, receiver operating characteristics, univariate and multivariate analyses, and subgroup analysis. The correlations between GIS and biological functions, glycolysis, immune activity, immune cell infiltration, and genomic changes were analyzed, and the potential of GIS to guide clinical treatment decisions was evaluated.

**Results:** A total of 54 prognostic GRGs and IRGs were identified, and a strong correlation was noted among them. However, most of them had somatic mutations and a high incidence of CNV. The GIS model that contained two GRGs (*PYGB* and *MDH1*) and three IRGs (*TSLP*, *SERPIND1*, and *GDF2*) was generated and a high GIS indicated poor survival. Moreover, we found that low GIS was associated with immune pathway activation, M1 macrophage infiltration, and higher immune scores. Finally, patients with low GIS were more sensitive to chemotherapy and immunotherapy.

**Conclusion:** An integrated model based on glycolysis and immune genes can distinguish the biological functions and immune infiltration patterns of individual tumors, quantitatively estimate the prognosis of patients with LUSC, and guide chemotherapy and immunotherapy decisions.

## Introduction

Lung cancer is one of the leading causes of cancer-related deaths worldwide and is the most common type of cancer (Siegel et al., 2020). Overall, 80%–85% of human lung cancers are non-small cell lung cancers (NSCLCs), and most NSCLCs contain two major histological subtypes, namely, lung adenocarcinoma (LUAD) and lung squamous cell carcinoma (LUSC), which account for approximately 25–30% of all lung cancers (Chen et al., 2014). Recent advances in targeted therapies have greatly benefitted patients with LUAD. However, little progress has been made in the development of LUSC-targeted therapies; as a result, traditional chemotherapy remains the first-line treatment of LUSC for decades. The 5-years survival rates of patients with advanced LUSC treated with currently used chemotherapy were less than 5% (Sun et al., 2007; Drilon et al., 2012), which appears overwhelmingly discouraging. Thus, there is an urgent need to determine prognostic biomarkers to identify patients who are sensitive to treatment. This will enable clinicians to predict clinical outcomes of LUSC timely and accurately and initiate personalized treatment regimens.

Abnormal tumor immune microenvironment (TIME) and tumor metabolic reprogramming are two important features of tumors (Hanahan and Weinberg, 2011). Cancer cells have traditional oxidative metabolism and glycolysis anaerobic metabolism. However, their proliferation is characterized by increased glycolysis metabolism, even in the presence of oxygen (Warburg effect) (Icard et al., 2018). Previous studies have focused on the Warburg effect, supporting the aggressiveness and drug resistance of cancer cells (Lu et al., 2015; Icard and Lincet, 2016), whereas the involvement of glycolysis and its product, lactic acid, in the regulation of TIME has recently become a research area. Studies have reported that lactic acid leads to tumor immune escape and inhibits the activity of T cells and natural killer (NK) cells while being up-taken by regulatory T (Treg) cells and maintaining their immunosuppressive ability (Brand et al., 2016; Watson et al., 2021). It can also inhibit monocyte activation and dendritic cell differentiation (Colegio et al., 2014). Moreover, it induces the M2 polarization of macrophages and promotes tumor growth through mechanisms by involving the hypoxia-inducible factor 1-alpha (HIF-1α) (Colegio et al., 2014). Although glycolysis has a clear inhibitory effect on the TIME, few studies have focused on this relationship comprehensively.

In this study, we integrated glycolysis-related genes (GRGs) and immune-related genes (IRGs) and constructed a systematic glycolysis–immune score (GIS) model. This GIS model showed stable prognostic efficacy in different datasets and clinical subgroups of LUSC. We also demonstrated the relationship of the GIS model to glycolysis and immune status and systematically explored the biological mechanisms of GIS from the perspectives of pathway activity, immune infiltration, and genomic changes. Finally, the study presents that GIS can identify patients with LUSC who are susceptible to chemotherapy and immunotherapy.

## Methods

### Genomic data and clinical information

RNA-sequencing data and clinical follow-up data from TCGA-LUSC patients were downloaded from the database of The Cancer Genome Atlas (TCGA). A total of 492 patients with LUSC were enrolled after excluding patients who had missing clinical information (such as stage, sex, and age) and who were lost to follow-up. In addition, three datasets, namely, GSE29013, GSE30219, and GSE37745, from the same chip platform (GPL570) were downloaded from the Gene Expression Omnibus (GEO) database. We enrolled patients whose pathological diagnosis was squamous cell carcinoma and excluded patients without detailed clinical information. Finally, 166 patients with LUSC were enrolled and used as a validation queue. The R Package ComBat was used to remove batch effects among datasets.

The corresponding MAF data of TCGA-LUSC patients on the Mutect2 platform were downloaded by the “TCGAbiolinks” package. Then, we used the R package maftools to process the MAF data, calculate the mutation load of samples, and draw the mutation map of genes.

Copy-number variation (CNV) data of patients were downloaded from the UCSC Xena Data Center (https://xena.ucsc.edu/) and preprocessed by GISTIC 2.0. Amplifications and deletions are defined with a threshold of 0.3.

The GRGs were collected from the MSIGDB database (www.gsea-msigdb.org), and the IRGs were collected from the ImmPort database (www.immport.org). The detailed gene list is provided in Supplementary Table S1.

### Construction of the GIS model

Initially, we screened the independent prognostic factors in GRGs and IRGs by univariate Cox regression. For significant independent prognostic factors (*p* < 0.05), we then used the random forest algorithm to identify the 10 most important prognostic genes within them. Then, we summarized all possible gene combinations of these 10 genes and determined the *p*-values of all combinations through Kaplan–Meier (KM) analysis. Based on the *p*-values, the gene combinations with the best prognostic efficiency were screened out. Then, the prognostic genes were used to construct a GIS model, as provided below:

$$GI\;Score=\sum iCoefficient\left({mRNA}_{i}\right)\times Expression\left({mRNA}_{i}\right)$$

The “servcomp” R package was used to calculate the consistency of the C index, and a larger C-index indicated that the prediction ability of the model was more accurate (Schröder et al., 2011). The high- and low-risk groups were divided based on the median GIS, and the prognostic value of the risk model was evaluated by the KM survival curve, univariate and multivariate Cox regression analyses, and time-dependent receiver operating characteristics (ROC) curve system.

### Functional enrichment analysis

The relative abundance of 22 immune cells per patient in the TCGA-LUSC cohort was calculated using the cibersortR package and LM22 feature. The ESTIMATE algorithm was used to calculate the immune score and matrix score of the samples. The R package gsva was used for single-sample gene set enrichment analysis (ssGSEA) to evaluate the pathway enrichment scores of the samples. The related pathway activity was collected from previously published references (Liberzon et al., 2011; Ayers et al., 2017; Gibbons and Creighton, 2018; McDermott et al., 2018; Liang et al., 2020). In addition, we collected the homologous recombination defect (HRD) score, neoantigens, and microsatellite instability (MSI) score (Thorsson et al., 2018) of samples from the study by Thorsson et al. to evaluate patient response to immunotherapy. Detailed gene sets are provided in Supplementary Table S2.

### Prediction of chemotherapy and immunotherapy responses

The R package “pRRophetic” can evaluate patients’ response to chemotherapy based on the Genomics of Drug Sensitivity in Cancer database. Five first-line agents for treating LUSC (namely, cisplatin, docetaxel, gemcitabine, paclitaxel, and vinorelbine) were selected, and the median maximum inhibitory concentration (IC50) for each patient was calculated using ridge regression to assess the sensitivity to chemotherapy in high- and low-risk groups. Then, the 10-fold cross-validation was used to enhance the predictive accuracy. The Tracking of Indels by Decomposition (TIDE) algorithm was used to assess patient response to anti-programmed death-1 (PD1) and anti-cytotoxic T-lymphocyte-associated protein 4 (CTLA4) therapy. Then, we matched the genome data of the high and low subgroups to a publicized cohort of 47 patients who can react to anti-PD1 and anti-CTLA4 therapy by using unsupervised subclass mapping (https://cloud.genepattern.org/gp/) and thus predict the response of high and low subtypes to immunotherapy.

Finally, we constructed a GIS model of a PD1-treated NSCLC cohort (GSE135222) and a mature PDL1-treated urothelial carcinoma cohort (IMvigor210) to evaluate the predictive power of GIS for immunotherapy response rates. GSE135222 included 27 patients with NSCLC treated with PD1, and the IMvigor210 cohort included 298 patients with melanoma treated with PDL1 and has integrated clinical information.

### Bioinformatics and statistical analysis

All statistical analyses and mappings were performed using R software version 4.04 (R Foundation for Statistical Computing, Vienna, Austria). The time-dependent area under the curve (AUC) was calculated using the R package “survivalROC” to evaluate the predictive power of variables. Univariate and multivariate COX regression analyses were performed using the R package “Survival.” A nomogram was drawn using the “rms” package. The R package “DCA” was used to draw decision curve analysis (DCA) curves. The Kruskal–Wallis test was used to compare more than two groups and the Wilcoxon test to compare two groups. The proportion differences were compared by the chi-square test. KM plotters were used to generate survival curves for subgroups in each dataset. Pearson correlation was used for correlation tests.

## Results

### Preliminary screening of prognostic GRGs and IRGs in LUSC

**FIGURE 1:**
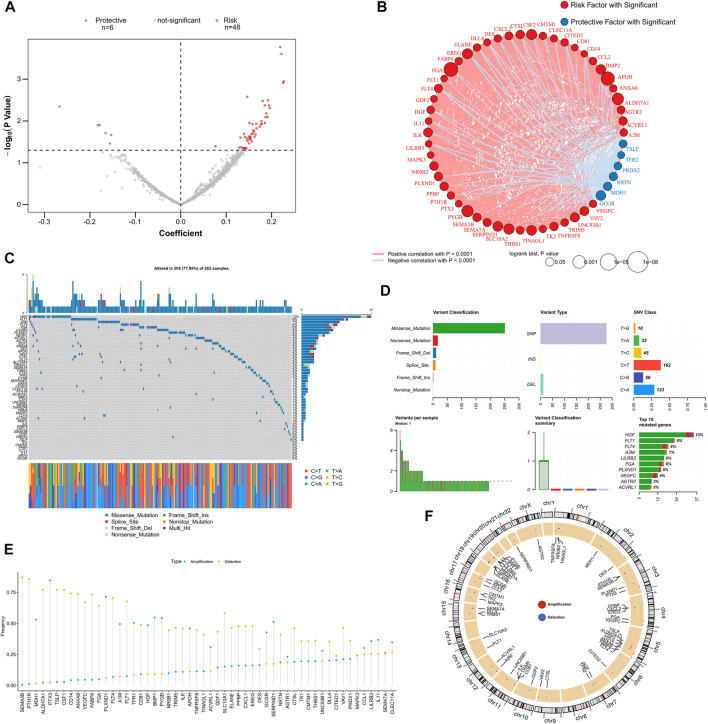
Identify prognostic-related GRGs and IRGs. **(A)** The volcano map illustrates the results of the univariate Cox analysis. **(B)** Correlation network of prognostic GRGs and IRGs. **(C)** Oncoplot displays mutation maps of prognostic GRGs and IRGs. **(D)** Summary of prognostic GRGs and IRGs mutation events in TCGA-LUSC. **(E)** Summary of CNV events for prognostic GRGs and IRGs in TCGA-LUSC. **(F)** The circle diagram presents the CNV maps of prognostic GRGs and IRGs on chromosomes. CNV, copy number variation; IRGs, immune-related genes; GRGs, glycolysis and immune score; TCGA-LUSC, The Cancer Genome Atlas Lung Squamous Cell Carcinoma.

We screened for independent prognostic factors in GRGs and IRGs in the training dataset (i.e., TCGA-LUSC cohort) and performed univariate Cox regression analysis to select genes that are significantly associated with prognosis. A total of 54 prognostic factors were identified, which included 48 risk factors and six protective factors (Figure 1A). Figure 1B displays their correlation network; six protective genes were negatively correlated with other genes, and 48 risk genes were positively correlated with other genes. Oncoplot presented mutation maps of prognostic factors in LUSC (Figure 1C). Specifically, the most common mutation of prognostic factors was a nonsense mutation, the most common change in base started from cytosine to thymine, and the *HGF* gene had the highest mutation frequency (Figure 1D). Fifty-four prognostic factors had extensive CNV events in LUSC (Figure 1E). The circle diagram presents their overall CNV status on chromosomes (Figure 1F). Most of the mutations in prognostic genes were nonsense mutations, whereas CNV events occurred extensively, suggesting that prognostic genes were mainly regulated by CNV than by single nucleotide variation.

### Generation and evaluation of GIS models

**FIGURE 2:**
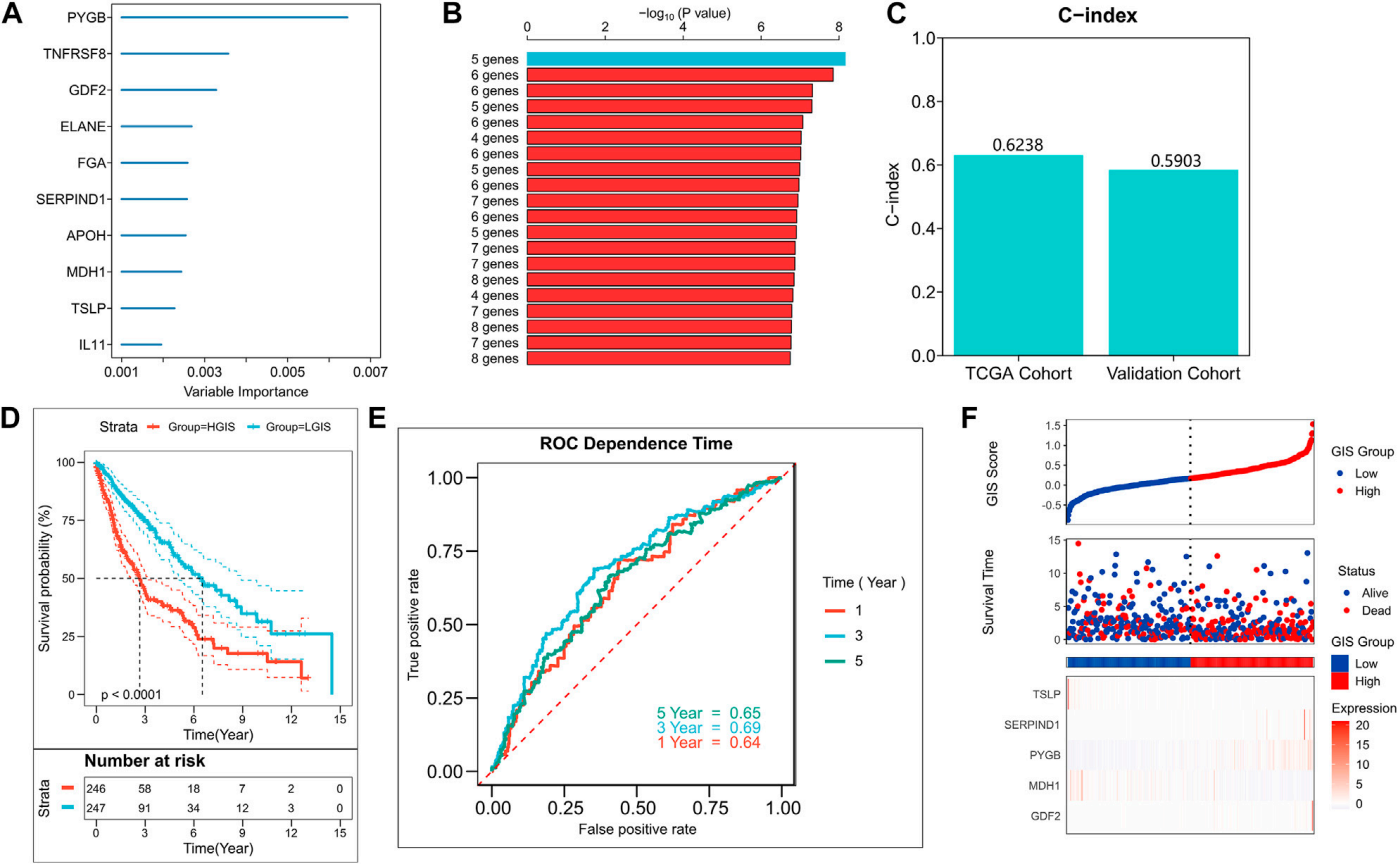
Construction of the GIS risk model. **(A)** Random forest screening of top10 important prognostic genes. **(B)** Log-rank test of *p*-value for each gene model. **(C)** C-index of the best model in TCGA and GEO queues. **(D)** KM survival curves of high and low GIS groups in the TCGA cohort. **(E)** ROC curves of GIS in the TCGA cohort at 1, 3, 5, and 8 years **(F)** Survival status and model gene expression of patients in the TCGA cohort. GEO, Gene Omnibus Expression; KM, Kaplan–Meier; ROC, receiver operating characteristics curve; GIS, glycolysis–immune score; TCGA, The Cancer Genome Atlas.

We used the random forest algorithm to identify the 10 most important genes among the 54 prognostic factors (Figure 2A). Then, we used the exhaustion method to find all combinations of the 10 genes and found 1,023 of them. A Cox regression model was constructed by gene combination, and the *p*-value of each model was evaluated by KM analysis. Finally, a five-gene model was selected to be the best prognostic model (Figure 2B), and detailed results are provided in Supplementary Table S3. The model contains two GRGs (*PYGB* and *MDH1*) and three IRGs (*TSLP, SERPIND1*, and *GDF2*), and the gene coefficients are listed in Supplementary Table S4. The C-index display model demonstrated good predictive performance in TCGA queues and external validation queues (Figure 2C). In the survival analysis, the survival rate of the high GIS group was significantly lower than that of the low GIS group (Figure 2D, *p* < 0.0001). The AUC values of the model at 1, 3, and 5 years were 0.64, 0.69, and 0.65, respectively (Figure 2E). Figure 2F presents the distribution of GIS in the TCGA cohort and the transcription map of the model genes. We also evaluated the effectiveness of GIS in an external validation queue. In the survival analysis, the survival of patients with high GIS was significantly worse (Supplementary Figure S1A, *p* = 0.013). In the ROC analysis, the AUC values of GIS in 1, 3, and 5 years were 0.61, 0.61, and 0.63, respectively (Supplementary Figure S1B). Supplementary Figure S1C illustrates the distribution of GIS in the GEO queue and the transcription map of the model genes.

### Evaluation of the predictive independence of GIS models

**FIGURE 3:**
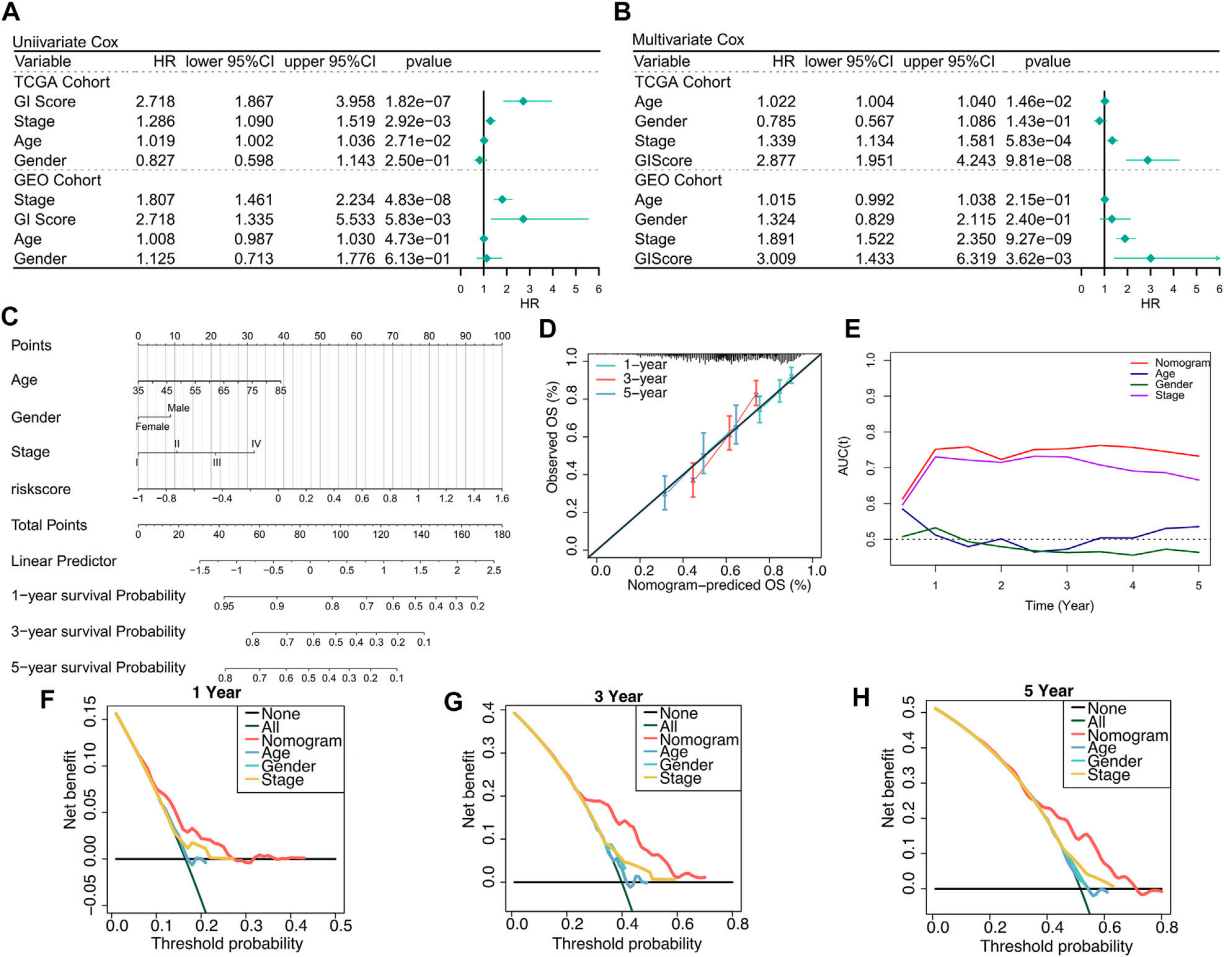
Verifying the GIS-related risk model. **(A)** Univariate Cox regression analysis of GIS and clinical features in the TCGA and GEO datasets. **(B)** Multivariate Cox regression analysis of GIS and clinical features in the TCGA and GEO datasets. **(C)** Nomogram based on the GIS model to quantify individual patient risk. **(D)** Nomogram calibration curve. **(E)** tROC curves of nomogram and clinical features. Nomogram DCA curves at 1 **(F)**, 3 **(G)**, and 5 **(H)** years. DCA, decision curve analysis; GEO, Gene Omnibus Expression; ROC, receiver operating characteristics curve; GIS, glycolysis–immune score; TCGA, The Cancer Genome Atlas.

We firstly used univariate Cox and multivariate Cox regressions to analyze the relationship between the risk score, clinical characteristics, and prognosis. Univariate Cox regression was an independent prognostic indicator in both training and validation sets (Figure 3A, *p* < 0.01). Multivariate Cox regression indicated that GIS was still an independent prognostic factor of overall survival in both training and validation cohorts after correcting other clinical features (Figure 3B, *p* < 0.01). The subgroup analysis also revealed that GIS remained a reliable prognostic factor in different clinical subgroups (Supplementary Figure S2). The GIS model proved to be a promising prognostic indicator for predicting the survival of patients with LUSC, and we subsequently constructed a nomogram to better quantify the risk assessment for these patients (Figure 3C). The nomogram correction curves reflected that the nomogram model had good stability and accuracy at 1, 3, and 5 years (Figure 3D). The TROC analysis revealed that the nomogram model was the best predictor when compared with clinical features (Figure 3E). We subsequently performed DCA to evaluate the decision benefits of the nomogram model and found that the nomogram is suitable for risk assessment of patients with LUSC at 1, 3, and 5 years (Figures 3F–H).

### Functional enrichment analysis and glycolysis spectrum of GIS

**FIGURE 4:**
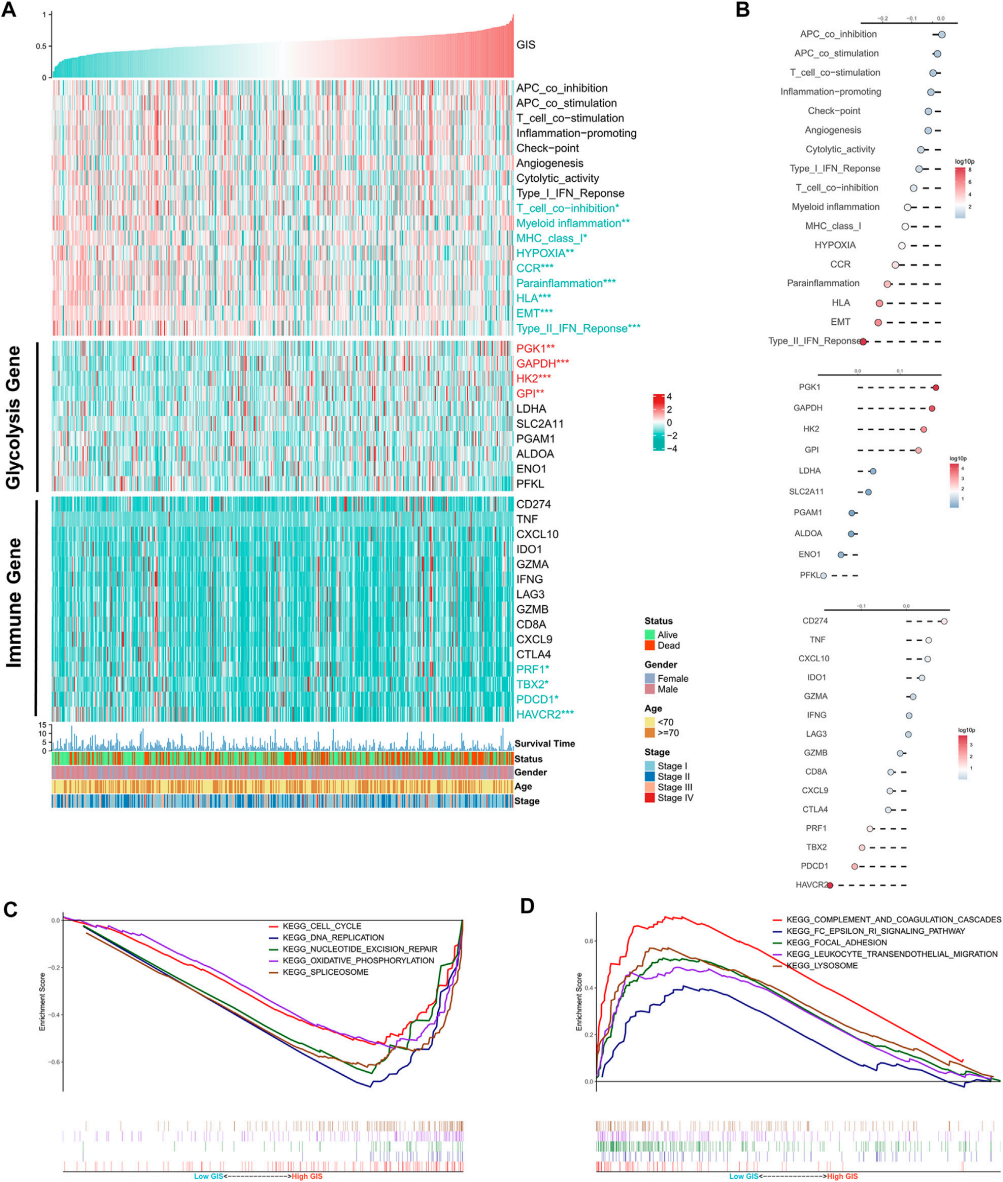
Functional analysis of the GIS risk model. **(A)** Heat maps of the correlations among GIS, biological pathway activity, glycolysis gene expression, immune gene expression, and clinical features. Red name with * represents upregulation in the high GIS group, and green name with * represents upregulation in the low GIS group; *p < 0.05, **p < 0.01, ***p < 0.001. **(B)** Correlation analysis of GIS and biological pathway activity, glycolysis gene expression, and immune gene expression (top to bottom). **(C)** GSEA enrichment map shows the five pathways of interest within the high GIS group. **(D)** GSEA enrichment map shows the five pathways of interest within the low GIS group. GIS, glycolysis–immune score; GSEA, gene set enrichment analysis.

Furthermore, we quantified the activity of some typical biological pathways using the ssGSEA algorithm and assessed the correlation between GIS and pathways. The heat map illustrates the relationship among GIS, biological pathway activity, classical glycolysis, and immune gene expression (Figure 4A). The corresponding correlation analysis is given on the right side of the heat map (Figure 4B). We found that EMT, hypoxia, and some immune-related pathways (such as the CCR, major histocompatibility complex [MHC] class 1, and type II interferon [IFN] response) GIS was significantly negatively correlated and significantly upregulated in the low GIS group. In addition, four glycolysis genes were positively correlated with GIS and upregulated in high GIS, whereas four immunity genes were negatively correlated with GIS and upregulated in low GIS. GSEA revealed that cell cycle, oxidative stress, and DNA replication activity were significantly increased in the high GIS group (Figure 4C), whereas lysosome and lymphocyte migration pathways were significantly enriched in the low GIS group (Figure 4D). In summary, these results suggest increased glycolysis activity and active tumor replication and proliferation in the high GIS group, whereas increased immune and cytotoxic activity in the low GIS group.

### Immune infiltration analysis of GIS

**FIGURE 5:**
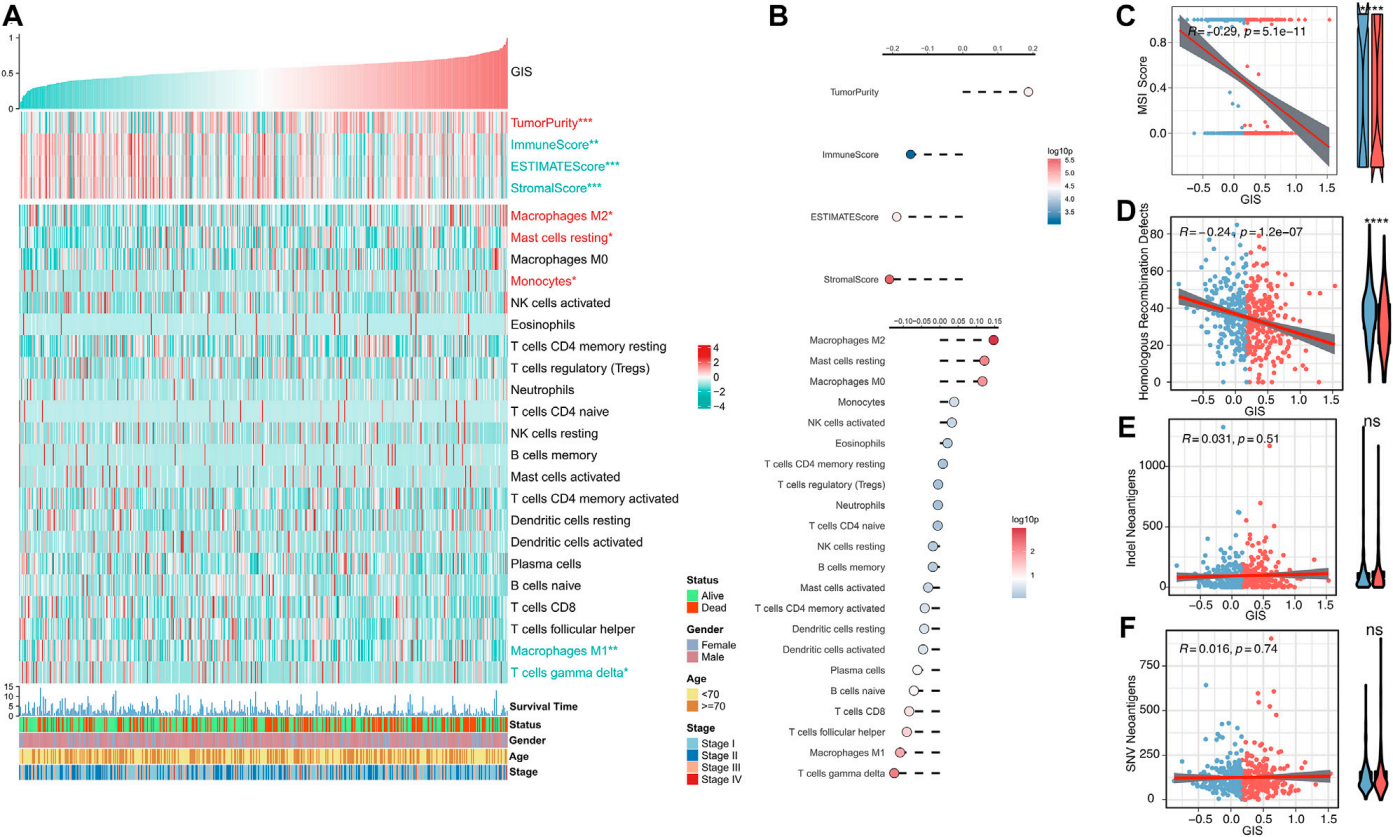
Immune landscape of the GIS risk model. **(A)** Heat maps of the correlations among GIS, estimate score, immune cell infiltration abundance, and clinical features. Red name with * represents upregulation in the high GIS group, and green name with * represents upregulation in the low GIS group; *p < 0.05, **p < 0.01, ***p < 0.001. **(B)** Correlation analysis of GIS, estimate score, and immune cell infiltration abundance (top to bottom). Scatter and box plots of the correlation between GIS and **(C)** MSI score, **(D)** HRD score, **(E)** indel neoantigens, and **(F)** SNV neoantigens. Indel, insertion and deletion; GIS, glycolysis–immune score; MSI, microsatellite instability; HRD, homologous recombination deficiency; SNV, single-nucleotide variant.

We further evaluated the correlation between GIS and immune landscape in detail. The heat map illustrates the correlation of GIS, estimate score, and abundance of immune-infiltrating cells (Figure 5A). The corresponding correlation analysis results are provided on the right side of the heat map (Figure 5B). The results revealed that patients with high GIS had higher tumor purity, whereas patients with low GIS had increased immune scores and estimate scores. Immune cell infiltration analysis also indicated that GIS was positively correlated with M2 macrophages and mast cells and increased in the high GIS group, whereas M1 macrophages and gamma delta T cells were negatively correlated with GIS and increased in the low GIS group. These results further suggest that antitumor immunity is suppressed in patients with high GIS, whereas antitumor immunity is active in patients with low GIS. Furthermore, we analyzed four indexes that affect the response to immunotherapy. Accordingly, the MSI and HRD scores were significantly negatively correlated with GIS and increased in the low GIS group (Figure 5C,D). This suggests that patients with low GIS have more chromosomal instability, leading to more tumor-specific neoantigen generation (Ganesh et al., 2019; Eso et al., 2020; Shi et al., 2021). However, no difference was found in insertion and deletion (indel) neoantigens and single-nucleotide variant (SNV) neoantigens between the high and low GIS groups (Figure 5E,F).

### Correlation between GIS and genome changes

**FIGURE 6:**
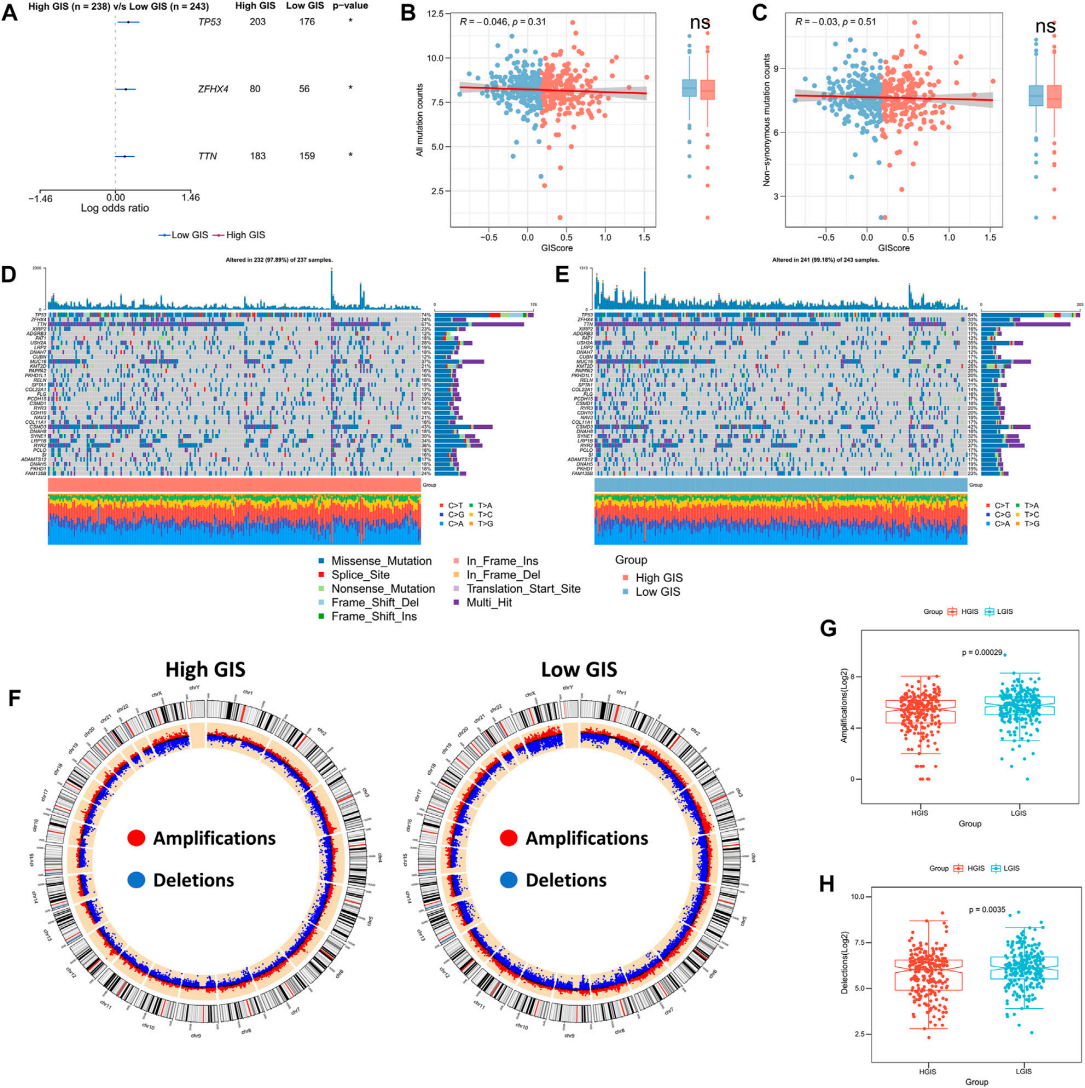
Genomic mutation landscapes of GIS models. **(A)** Forest map of the high-frequency mutated genes with significant mutation differences between the high GIS and low GIS groups. **(B)** Correlation between GIS and all mutant loads. **(C)** Correlation between GIS and non-synonymous mutation load. **(D)** Oncoplot of the high-frequency mutated genes in the high GIS group. **(E)** Oncoplot of the high-frequency mutated genes in the low GIS group. **(F)** Circle diagram summarizing CNV events on different chromosome arms in the high and low GIS groups. **(G)** Box plot of the difference in chromosome amplification between the high GIS and low GIS groups. **(H)** Box plot of the difference in chromosome deletions between the high GIS group and the low GIS group. GIS, glycolysis–immune score; CNV, copy number variation.

Recent studies have proposed using the tumor mutation burden (TMB) as a novel indicator in predicting immunotherapy response and prognosis, as more mutated genes may generate new antigenic peptides that can be recognized by the immune system. Antigens containing mutated peptides can activate the immune system and enhance anti-tumor immunity (Matsushita et al., 2012; Rizvi et al., 2015; Chan et al., 2019). Therefore, we explore the correlation between TMB and GIS. Through Fisher’s test, we identified three high-frequency mutated genes with significant mutation differences, namely, *TP53*, *ZFHX4,* and *TTN*, with increased mutation frequency in the low GIS group (Figure 6A). However, the number of mutation techniques and non-synonymous mutations demonstrated an increasing trend in the low GIS group, but it was not significant (Figures 6B,C). The waterfall diagram illustrates the mutation maps of high-frequency mutated genes in the high and low GIS groups (Figures 6D,E). CNV caused genomic changes in patients as chromosome segment changes, and we subsequently analyzed the correlation between CNV and GIS. The circle graph presents the overall CNV landscape of patients with high and low GIS, and the results revealed that patients with low GIS have more CNV events (Figure 6F). The box plot illustrates that both amplification and missing events in the low GIS group were significantly higher than those in the high GIS group (Figures 6G,H).

### The GIS model can guide clinical treatment decision

**FIGURE 7:**
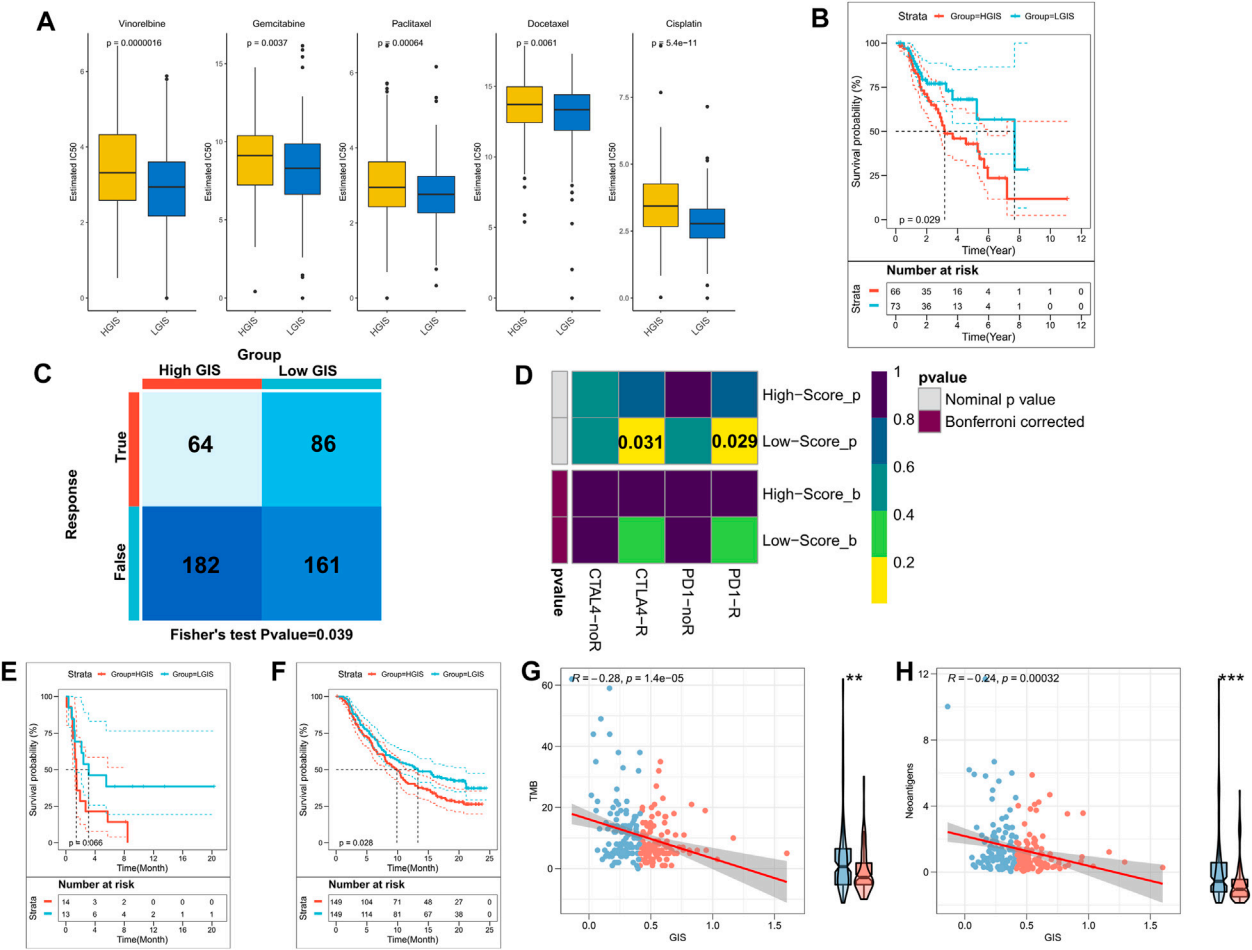
The GIS model guides the clinical treatment decision. **(A)** Box plot of the predicted IC50 values of five commonly used lung cancer drugs in the high and low GIS groups. **(B)** KM survival curves of patients receiving chemotherapy in the TCGA cohorts with high and low GIS. **(C)** The TIDE algorithm was used to predict the overall response rate to immunotherapy in the high and low GIS groups. **(D)** Subclass mapping was used to predict the sensitivity of patients in the high and low GIS groups to anti-PD1 and anti-CTLA4 therapy. **(E)** KM survival curves of high and low GIS groups in the GSE135222 cohort. **(F)** KM survival curves of the high and low GIS groups in the IMvigor210 cohort. **(G)** Correlation between GIS and TMB in the IMvigor210 cohort. **(H)** Correlation between GIS and neoantigens in the IMvigor210 cohort. GIS, glycolysis–immune score; GSEA, gene set enrichment analysis; TMB, tumor mutation burden; KM, Kaplan–Meier; TCGA, The Cancer Genome Atlas; TIDE, Tracking of Indels by Decomposition.

We firstly assessed the sensitivity of patients to five commonly used chemotherapy agents for lung cancer, namely, cisplatin, docetaxel, gemcitabine, paclitaxel, and vinorelbine. Accordingly, patients with low GIS were more sensitive to these five chemotherapeutic agents (Figure 7A). In the validation cohort, the low GIS group was more sensitive to the other four drugs, except for gemcitabine (Supplementary Figure S1D). The survival analysis revealed that among patients receiving chemotherapy in the TCGA cohort, survival was better in patients with low GIS (Figure 7B, *p* = 0.029). Previous results suggested that patients with low GIS may be more sensitive to immunotherapy; thus, we subsequently assessed patient response to immunotherapy. In the TIDE analysis, patients with low GIS were more sensitive to immunotherapy (Figure 7C), although not significant in the validation cohort (Supplementary Figure S1E). Subclass mapping indicated that patients with low GIS were more sensitive to anti-PD-1 and anti-CTLA-4 therapy, and the same results were observed in the validation cohort (Figure 7D, Supplementary Figure S1F). Subsequently, we validated GIS in an NSCLC cohort that received anti-PD-1 therapy, and the results presented poorer survival in patients with high GIS (Figure 7E, *p* = 0.066). The efficacy of GIS was also evaluated in IMvigor210, a large immunotherapy cohort, which exhibited significantly worse survival in patients with high GIS (Figure 7F). Further analysis revealed that TMB and neoantigens were negatively correlated with GIS in the IMvigor210 cohort and significantly increased in the low GIS group (Figures 7G,H). This may explain the high benefit of immunotherapy in patients with low GIS.

## Discussion

With the limited success of LUSC-related targeted therapies, traditional chemotherapy remains the first-line treatment for decades; thus, patients with advanced LUSC treated with current chemotherapy show poor 5-years survival rates, that is, less than 5%. Therefore, there is an urgent need to identify prognostic biomarkers to accurately and timely predict clinical outcomes of LUSC and initiate personalized treatment programs. Glycolysis not only plays an important role in tumor invasion and drug resistance but also has a strong inhibitory effect on the TIME (Brand et al., 2016; Watson et al., 2021). The complex role of glycolysis and TIME reflects great promise in immunotherapy and targeted cancer therapy (Ganapathy-Kanniappan and Geschwind, 2013; Ganapathy-Kanniappan, 2017). In this study, we constructed a GIS model based on GRGs and IRGs and demonstrated that this model has satisfactory predictive efficacy in different clinical subgroups of datasets. Therefore, it can be used as an independent prognostic factor for patients with LUSC. Furthermore, we explored the relationship between the GIS model and biological function, immune cell infiltration, and genome changes. Several transcriptomic models are proved to have promising applications in lung cancer and have surprising potential in predicting prognosis (Wang et al., 2021a; Gao et al., 2021; Feng et al., 2022; Guo et al., 2022; Jiang et al., 2022). Compared with these models, our model not only has good performance in predicting prognosis but also can distinguish between “cold” and “hot” tumors and provide a reference for clinical treatment decisions of patients with LUSC.

Immunotherapy has developed rapidly in LUSC in recent years (Lazzari et al., 2017). LUSC tends to be highly immunogenic and has higher TMB. Therefore, LUSC is an ideal indication for immunotherapy (Li et al., 2018). However, the overall response rate to immunotherapy is relatively low, and only a subset of patients with LUSC can benefit from immunotherapy (Forde et al., 2018). Therefore, the identification of patients with LUSC having “hot” tumors is expected to enhance treatment response to immunotherapy. Through functional enrichment analysis, we found that low GIS was associated with increased activity of some immune-related pathways, such as CCR, MHC class 1, and type II IFN response, and lysosome and lymphocyte migration, suggesting that the low GIS group was a “hot” tumor with anti-tumor immunoactivity (Ivashkiv, 2018; Dersh et al., 2021). We also analyzed the immune cell infiltration in the low GIS group and we found that the low GIS group had higher immune scores and increased M1 macrophages and gamma delta T cells, suggesting that low GIS tumors are immuno-activated “hot” tumors with antitumor activity (Chanmee et al., 2014; Kabelitz et al., 2020; Yazdanifar et al., 2020). The cell cycle and DNA replication pathway were enriched in the high GIS group, indicating that tumor proliferation was active in this group. Furthermore, oxidative stress activity increased in the high GIS group, and oxidative stress stimulates tumorigenesis and supports tumor cell proliferation (Hayes et al., 2020; Kotsafti et al., 2020). Moreover, high GIS was associated with increased glycolysis activity, and low GIS was associated with increased immune gene activity. Furthermore, we analyzed the immune cell infiltration in the TIME of high GIS group, and the results revealed that a high GIS was associated with higher tumor purity and M2 macrophages, which may lead to immunosuppression and tumor-promoting TIME (Chanmee et al., 2014) in the high GIS group. These results suggest that high GIS could identify patients with “cold” tumors, high glycolysis, metabolically active tumors, and suppressed antitumor immunity. Subsequently, we found that the HRD and MSI scores were negatively correlated with GIS and significantly increased in the low GIS group, indicating that tumors with low GIS may be more sensitive to chemotherapy, have high immunogenicity, and are more sensitive to immunotherapy (Le et al., 2017; Overman et al., 2017; Hoppe et al., 2018; Silva et al., 2022). However, no significant difference was found in the number of neoantigens between the two groups.

We subsequently found that *TP53*, *ZFHX4,* and *TTN* mutated more frequently in the low GIS group. *TP53* is generally considered a tumor-suppressor gene (Bykov et al., 2018; Skoulidis and Heymach, 2019), whereas the *TP53* gene in the low GIS group shows a better survival rate and more mutations, which may be caused by the active immune function of low GIS. Recent studies have reported that genomic changes are closely related to neoantigen formation and immunotherapy response (Anagnostou et al., 2017). Our results indicate that TMB differences between low and high GIS groups are not significant, and GIS can better reflect patients’ immune activity than TMB. We also found that both CNV amplification and deletion events were significantly higher in the low GIS group, and the chromosomal changes were more closely related to GIS than the single nucleotide variation. Studies have shown that chromosomal somatic rearrangement events actively promote carcinogenesis and lead to immunosuppression. However, our analysis showed that immunoactivity was stronger in the low GIS group than in the high GIS group. These results suggest that GIS can better reflect tumor immune status and predict immunotherapy response than TMB and CNV.

In summary, low GIS appears to indicate “hot” tumors with an immunoactivated phenotype that may be more sensitive to treatment. We then systematically assessed patient response to chemotherapy and immunotherapy. Accordingly, we found that the low GIS group was more sensitive to chemotherapy than the high GIS group. In addition, TIDE and subclass mapping algorithms predicted that patients with low GIS would be more sensitive to immunotherapy. More convincingly, we found that a low GIS was associated with better outcomes in the immunotherapy cohort of NSCLC. In a further large-scale immunotherapy cohort, IMvigor210, better survival was observed in patients with low GIS. A negative correlation was noted between GIS and TMB and neoantigens in the IMvigor210 cohort. Immunotherapy mainly relies on CD8^+^ T cells to recognize tumor-specific mutant antigens to induce antitumor immunity (Wang et al., 2021b; Jhunjhunwala et al., 2021). In addition, more somatic mutations will lead to the formation of more potential new antigens (Matsushita et al., 2012; Rizvi et al., 2015). Therefore, more neoantigens and TMB in the low GIS group may lead to the increased sensitivity of patients with low GIS to immunotherapy. Taken together, these results demonstrate that the GIS model is a powerful tool for guiding the treatment of patients with LUSC and that patients with low GIS have a higher sensitivity to chemotherapy and immunotherapy.

Despite its findings, this study has some limitations. First, this study was based on high-throughput sequencing and only considered inter-patient heterogeneity, but there was no intra-tumor heterogeneity. Second, immunotherapy and chemotherapy sensitivity predictions are based on computations and should be validated in further clinical cohorts. Thus, additional *in vivo* and *in vitro* experiments are needed to explore the specific biological functions of GIS in LUSC.

In conclusion, the results of this study suggest a close relationship between glycolysis and immune activity. Moreover, the integrated model based on glycolysis and immune genes can distinguish “cold and hot” patterns of individual tumors from biological function and immune infiltrating system, can quantitatively estimate the prognosis of patients LUSC, and guide chemotherapy and immunotherapy decisions.We thank all the participants who supported our study. In particular, thanks to the TCGA database and GEO database for the analytical data.

## Data Availability

The raw data mentioned in this study can be downloaded from online databases. More detailed information can be provided by the corresponding author upon reasonable request.

## Supplementary material

Supplementary Figure S1 External validation of GIS **(A)** KM survival curves of high and low GIS groups in the GEO cohort. **(B)** ROC curves of GIS in the GEO cohort at 1, 3, 5, and 8 years **(C)** GIS, survival status, and model gene expression profile of patients in the GEO cohort. **(D)** Box plot of the predicted IC50 values of five commonly used lung cancer drugs in the high and low GIS groups of the GEO cohort. **(E)** The TIDE algorithm was used to predict the response of high and low GIS groups to immunotherapy in the GEO cohort. **(F)** Subclass mapping was used to predict the sensitivity of patients in the high and low GIS groups to anti-PD1 and anti-CTLA4 treatment in the GEO cohort. GIS, glycolysis–immune score; KM, Kaplan–Meier; GEO, Gene Omnibus Expression; TIDE, Tracking of Indels by Decomposition; PD1, programmed death-1 (PD1); CTLA4, anti-cytotoxic T-lymphocyte-associated protein 4.

Supplementary Figure S2 Subgroup Cox analysis of GIS Subgroup Cox regression analysis of GIS in the TCGA queue (A) and GEO queue (B). GIS, glycolysis–immune score; GEO, Gene Omnibus Expression; TCGA, The Cancer Genome Atlas.
